# Some Account of a Recent French Publication, Respecting the Pestilential and Malignant Fevers of the Levant

**Published:** 1802-11-01

**Authors:** R. Hall

**Affiliations:** Pancras


### *Some Account of a recent French Publication, respecting the Pestilential and Malignant Fevers of the Levant; communicated by Dr.* HALL, *of Pancras.*

THE public has of late been favoured with many judicious observations on pestilential fevers, as well as other diseases, which are more particularly prevalent in warm climates. As this subject, however, is by no means exhausted, and cannot fail to prove highly interesting to such a commercial nation as Great Britain, the present Work will, we doubt not, although not adding much to our present stock of information, be yet considered as furnishing an article worthy of insertion in the Medical and Physical Journal.

Dr. Pugnet, after presenting his readers with a short account of the temperature, the soil, and the diseases of Egypt, proceeds to the consideration of the plague, and other malignant fevers, which more particularly fell under his own observation, during the continuance of the French army in that country and Syria.

In Egypt the hot season occupies one half of the year, extending from the middle of March to the beginning of September, or from the vernal to the autumnal equinox. This period is divided by our author into what he terms the first and second summer. During the former period, the air is either extremely hot, and loaded with humidity, or it is dry, and charged with a thick cloud of very fine dust, according to the prevalence of the south-east or south-west wind; during the latter, the purity and salubrity of the air are restored, and the heat moderated by winds from the north, or north-east. The rainy rainy feafon commences in autumn, and is fometimes accompanied with terrible thunder-ftorms. But of all the feafons in Egypt, the winter is that which is the moft agreeable; the fky is clear, the heat moderate, and vegetation, which is never wholly interrupted, now proceeds with increafed vigour.

Befides the difeafes of more temperate regions, all of which are known in Egypt, and efpecially thofe depending on a difeafed ftate of the lymphatic fyftem, our author mentions others, which may be faid more particularly to belong to that climate. Among thefe we find the plague, and the *Dem-el-monia*, an account of which forms no inconfiderable portion of the prefent volume; as well as the *Demaouïe*, which, from the defcription given of it, appears to refemble that fpecies or variety of apoplexy, termed by Sauvages Carus ab infolatione.

Dyfenteries are alfo extremely prevalent in this country during the fecond fummer; they affume a putrid type, and are fometimes accompanied with a continued, fometimes with a remittent fever.

The inhabitants of the Saïd are not much fubject to difeafes of the fkin, but in Lower Egypt, and along the coaft, we are informed the elephantiafis and other cutaneous diforders are not unfrequent. Dr. P. particularly defcribes a very troublefome erythematous affection, which makes its appearance during the courfe of the fecond fummer, and which he imputes to the faline particles with which their waters are abundantly impregnated.

Ophthalmia is likewife much more formidable in Lower than in Upper Egypt; the fymptoms, as here enumerated, do not differ effentially from other accounts which have already been given of this difeafe; but, in our opinion, the treatment was not the moft judicious or effectual that might have been purfued; for although it appears that the inflammation was extremely violent, neither blifters, fcarifications of the eye itfelf, nor topical bleeding, under any mode whatever, were employed.

The author next proceeds, at fome length, to fhow in what fenfe the plague may be termed endemic in Egypt, and to point out thofe meafures which, in his opinion, are neceffary to be purfued, in order to exterminate the exifting contagion. He likewife propofes feveral additional articles to the code of health, refpecting the regulations in Lazarettos, and the performance of quarantine; but as thefe do not appear to be either novel, or very important, we fhall pafs them over, with obferving that we were rather furprifed to find our author has either omitted to mention, or been wholly unacquainted with the important procefs firft recommended by Dr. Johnftone, and fince fo fuccefsfully cefsfully employed by his countryman Guyton, for the correction and deftruction of peftilential virus.

Dr. P. now gives a defcription of the epidemic which prevailed in the French army during its continuance in Syria, which does not however appear materially to differ from that given by other writers. He divides it into three fpecies; inflammatory, putrid, and nervous; or according as they differ from each other, in being attended with increafed activity in the vafcular fyftem, with fymptoms of debility, or with thofe which denote a tendency of the fluids to putrefaction.

Without entering into any difquifition refpecting the nature of this contagion, our author only obferves that all thefe fpecies were propagated by infection, and adduces feveral facts which fell under his own obfervation in confirmation of this opinion. Eight Frenchmen at Caipha were fucceffively affected with the difeafe, by conveying a peliffe to each other; five or fix, at Gaza, by difputing for the caft-off clothes of one of their companions; and four, at Jaffa, in confequence of wearing the neckcloths of an apothecary, who had died of the plague. Thefe laft were affected with buboes round the neck, and fell victims to the difeafe between the third and fixth day.

Some late writers have, we are aware, endeavoured to controvert this doctrine, becaufe a few individuals, who maintained a direct communication with the fick, efcaped with impunity; but it furely does not thence follow, that this epidemic is not of a contagious character, fince, unlefs the neceffary predifpofition concur with the application of the exciting caufe, it muft ever fail to produce the difeafe. Examples of a fimilar exemption are not unfrequent on expofure to the venereal and variolous poifons, which are yet univerfally allowed to be contagious: befides, on that principle, as Dr. P. juftly obferves, how fhall we explain the fafety of thofe who remained in a ftate of feclufion, during the prevalence of this epidemic peftilence? The particular ftate of the French army, both in Syria and at Damietta, prevented our author from fully afcertaining many facts refpecting the mode in which this contagion was propagated: he agrees however with moft writers on this fubject, that actual contact with infected perfons, or things, is neceffary to produce the difeafe; or, at leaft, that the contagious effluvia act only near to the fources whence they arife. Many circumftances, however, induced him to believe that patients labouring under this epidemic ceafed to communicate the difeafe, as foon as the fever was extinguifhed, whatever might be the ftate of the buboes or carbuncles at that period. But we regret he did not endeavour to afcertain what interval of time elapfes between expofure to the contagion, and the firft appearance appearance of the fever; or, in other words, how long the poison remains dormant in the system; since, as Dr. Haygarth justly observes, on the determination of this point, the rules of quarantine ought to be formed.

Various causes, it is well known, predispose, or render the body more liable to be affected by the operations of contagion. Whilst Dr. P. enumerates many of these causes, and points out the necessity of avoiding them, he at the same time recommends the employment of such measures as appear to him proper to strengthen the constitution, and enable it to resist the influence of pestilential contagion.

In this part of the work, we find a few remarks on the prophylaxis. Every attention, which circumstances could possibly admit, seems not only to have been paid to obtain ventilation and cleanliness, but to prevent all intercourse between the diseased and healthy, by the removal of every person, so soon as any symptoms of infection appeared, to the common Lazaretto.

Our author, after presenting his readers with a pretty full statement of the favourable and unfavourable appearances of this disorder, as it prevailed among the French army in Syria and at Damietta, proceeds to a detail of its medical treatment, which appears, on the whole, to have been judicious, or not to differ very materially from that now generally adopted. If, however, it seems less marked by decision and vigour than could be wished, great allowances must be made for the peculiar situation of the author, during the course of an irregular warfare, and not only without the necessary assistants, but destitute of almost every requisite medicine. We find him even reduced to the necessity of supplying the want of vesicatories, by boiling water or vinegar, or by the application of the actual cautery. These, as well as other circumstances, joined to the want of a tabular view of the number of patients received into the hospitals, and the proportion cured, render it difficult fully to appreciate the merit of Dr. Pugnet's practice.

With respect to the pestilential malady which prevailed among the French troops at Cairo, as it appears to have in no respect differed from that described in the former part of this volume, we shall not detain our readers with any account of it, but proceed to notice the *Dem-el-monia*, which is the last subject that engages our author's attention in the present publication.

From the description given of this malady, it evidently appears to be an intermittent fever of the tertian form, which, unless vigorous measures be employed to arrest its progress, speedily

ſpeedily proves fatal. In combating this affection, our author ſeems very properly to have placed his chief reliance on the liberal exhibition of the Peruvian bark, during the period of intermiſſion, or apyrexia. But he appears to heſitate with reſpect to the proper time of adminiſtering that medicine, when this fever aſſumes a remittent or continued type.

Every practitioner, however, who has carefully attended to the nature and ſymptoms of ſuch fevers, and witneſſed their rapid progreſs to a termination in a warm climate, muſt acknowledge, that whenever the remiſſions are inconſiderable, or great danger is apprehended from repeated paroxyſms, the bark ought to be preſcribed, not only without any regard whatever either to remiſſions or exacerbations, but in as large doſes as the ſtate of the patient's ſtomach will allow.

